# Influence of Finer Ceramic Roof-Tile Waste Powder as a Cement Substitute on Mortar Properties

**DOI:** 10.3390/ma19102124

**Published:** 2026-05-19

**Authors:** Agus Maryoto, Nor Intang Setyo Hermanto, Voilla Oktaviani

**Affiliations:** Department of Civil Engineering, Jenderal Soedirman University, Jl. Mayjen Sungkono KM 5, Blater, Purbalingga 53371, Indonesia; nor.hermanto@unsoed.ac.id (N.I.S.H.); voilla.oktaviani@mhs.unsoed.ac.id (V.O.)

**Keywords:** cement substitution, ceramic roof tile, fineness, mortar, workability

## Abstract

The increasing demand for sustainable construction materials has intensified interest in reusing ceramic waste as a supplementary material in cementitious systems due to its potential to reduce environmental impacts and enhance resource efficiency. Previous studies indicated that ceramic roof tile waste powder (CTP) with a fineness value greater than that of cement did not contribute to an enhancement in the compressive strength of mortar. This study investigates CTP with a higher fineness than cement. Experimental parameters include fineness analysis, mortar flow, setting time, and compressive strength test. The instruments used are the Blaine tools for fineness testing, the flow table for mortar flow testing, the Vicat tools for setting time testing, and the Mortar Compression Machine for compressive strength testing. Mortar specimens (5 × 5 × 5 cm^3^) were prepared by partially replacing cement with CTP at different substitution levels. The results indicate that the addition of finely ground CTP increases mortar flow, extends setting time, and enhances compressive strength, suggesting its potential as a supplementary cementitious material in mortar applications.

## 1. Introduction

The waste materials have become common constituents in various cementitious composites, including concrete and mortar, as part of broader efforts to enhance sustainability in the construction industry. The study utilizes waste materials that originate either from routine community use or from industrial production processes that yield a considerable proportion of defective products. The materials used in this study comprise waste products commonly generated from everyday human activities.

A growing body of research indicates that industrial waste materials can serve as effective partial substitutes in cementitious systems. They are offering measurable reductions in environmental impact within the context of sustainable development. Studies assessing waste-based binders for wall plaster have shown that conventional production methods contribute substantially to environmental burdens. On the other hand, green mortar alternatives provide pathways to mitigate these impacts while improving thermal performance and reducing energy consumption. Collectively, prior investigations underscore the potential of incorporating industrial by-products into mortar mixtures as a viable strategy. Waste usage is conducted to advance sustainability objectives in the construction sector [1].

Previous studies have investigated the use of waste materials as substitutes in mortar and cement mixtures. The waste-based substitution materials examined in these studies include egg shell powder, rice husk ash, waste brick powder, and ceramic tile waste [2,3,4,5]. These waste-derived materials are processed to achieve physical and mechanical properties comparable to those of the targeted substitution materials. However, waste-related issues in mineral-based materials such as ceramics and slate remain a significant challenge, primarily due to conventional manufacturing and drilling processes that can induce material damage. Therefore, technological innovations such as CO_2_ laser processing have been developed to enhance efficiency and minimize such damage. Previous studies have demonstrated that this method is capable of producing micro-holes without inducing cracks, thereby reducing production waste [6].

The use of construction and demolition waste (CDW) as recycled aggregates in mortar shows that mechanical performance depends on the material type and its crystalline phase composition. Aggregates from concrete, natural stone, and ceramics perform better than those from brick and roof tiles, while mixing with natural aggregates further improves performance. Therefore, proper phase characterization and homogeneous separation of CDW are essential for achieving stable and predictable material properties [7].

In a previous study, the mechanical performance of ceramic waste incorporated into concrete was assessed using replacement levels of 0%, 10%, 20%, 30%, 40%, and 50%, resulting in eleven mix compositions. The results demonstrated that ceramic waste functions more effectively as a fine aggregate replacement than as a coarse aggregate substitute, yielding superior strength characteristics [8].

Several studies have also explored the utilization of ceramic roof-tile waste as aggregate. One investigated the use of crushed roof-tile waste as a fine aggregate replacement [9], and another examined the influence of large quantities of roof-tile waste used as coarse aggregate [10]. And others evaluated the performance of self-compacting concrete incorporating recycled traditional roof-tile powder [11]. Overall, these studies indicate that ceramic roof-tile waste can serve as an effective substitute for both fine and coarse aggregates.

Ceramic roof tiles are building components used as roofing materials, manufactured from clay—with or without additional constituents—and fired at high temperatures, as specified in the Indonesian roofing standard [12]. Ceramic roof tiles are widely utilized in Indonesia due to their affordability and their favorable thermal and acoustic performance.

The manufacturing of ceramic roof tiles is generally carried out by skilled producers, where a production volume of around 20,000 tiles typically results in approximately 95% meeting standard quality specifications, with only about 5% categorized as defective [13]. The defective tile rate of approximately 5–25% constitutes a significant waste volume when compared with the annual output of the ceramic roofing industry, particularly the Sokka tile sector in Kebumen. In 2010, regional production reached 174.424.333 units (≈305,242 tons) [14]. Even at a conservative defect rate of 5%, the industry generates an estimated 15,262 tons of ceramic tile waste annually.

The author observes that research on the utilization of ceramic waste generated from tile production remains limited. A study conducted in 2024 investigated the use of ceramic roof-tile waste as a cement substitute, employing a powder fineness of 2753 cm^2^/g, which is lower than the grain size of ordinary cement. The results indicated an increase in both initial and final setting times at replacement levels of 0%, 10%, 20%, 30%, and 50% [15]. Another study [16] reported that incorporating ceramic tile powder (CTP) at replacement levels of up to 20% can still produce mortar with strength properties comparable to the control mixture, whereas higher substitution ratios lead to significant strength degradation. The integration of CTP as a partial cement replacement is considered strategically beneficial, as it can reduce cement consumption while simultaneously minimizing the amount of ceramic tile waste disposed of in landfills.

Existing literature indicates that studies utilizing ceramic roof-tile waste as either a partial or complete cement replacement in concrete are still scarce. In the present work, CTP will be incorporated at fineness levels comparable to ordinary Portland cement and in accordance with the Indonesian Standard SNI 15-2049-2004 [17], which requires a minimum specific surface area of 2800 cm^2^/g. The substitute material will be evaluated at replacement proportions of 0%, 10%, 20%, 30%, and 50%.

## 2. Materials and Methods

This paper was performed through a laboratory-based experimental program. The workflow encompassed literature assessment, preparation and calibration of equipment, characterization of raw materials, fabrication of mortar specimens, execution of mechanical and physical tests, and subsequent data interpretation. The materials utilized in this study consisted of PCC (Portland Composite Cement, PT. Solusi Bangun Indonesia Tbk, Cilacap, Indonesia), distilled water, silica sand, and finely processed ceramic roof-tile waste. The testing apparatus included crushing and milling equipment, an oven, a sieve series, a Blaine fineness device, a flow table, and a mortar mixer.

A total of fifteen defective ceramic roof tiles sourced from a local manufacturer served as the waste precursor. The tiles were subjected to sequential size-reduction and comminution processes to obtain a powder suitable for incorporation into mortar mixtures. The Portland Composite Cement presented a specific surface area of 4677 cm^2^/g.

**Figure 1:**
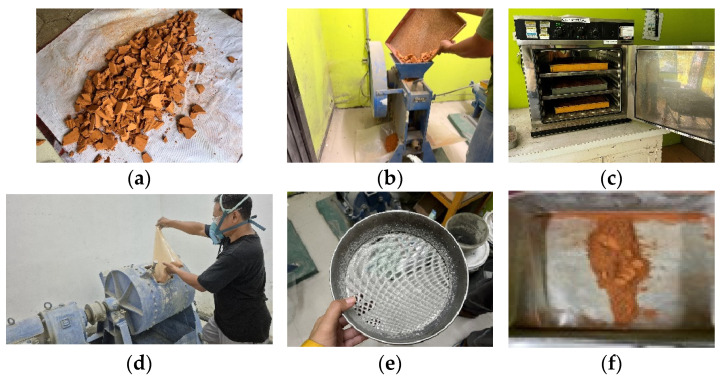
A sequence of processing stages for Sokka roof tile waste to produce a fine powder material before testing. (**a**) fragment of ceramic roof-tile waste; (**b**) reducing size by Jaw crusher; (**c**) oven; (**d**) Finish mill; (**e**) Siever no. 280; (**f**) fine powder of CTP.

Figure 1 summarizes the preparation protocol. Figure 1a, the roof tiles were air-dried for two days to reduce inherent moisture and subsequently broken into 3–5 cm fragments using manual impact. In Figure 1b, fragments underwent primary crushing via a jaw crusher to achieve smaller particle sizes. The crushed material was then oven-dried at 100 °C for 24 h to eliminate residual moisture at Figure 1c. Figure 1d–f grinding was performed using a finish mill charged with steel grinding media (30–80 mm diameter), which facilitated particle breakage through impact and abrasion mechanisms. The milled product was finally classified using a No. 280 sieve to obtain a uniform fine powder appropriate for mortar production.

Mortar specimens incorporating CTP as a partial cement replacement were evaluated. Five replacement ratios were investigated: 0% (control), 10%, 20%, 30%, and 50%. The detailed mixture of the mortar investigated in this study is shown in Table 1.

**Figure 2:**
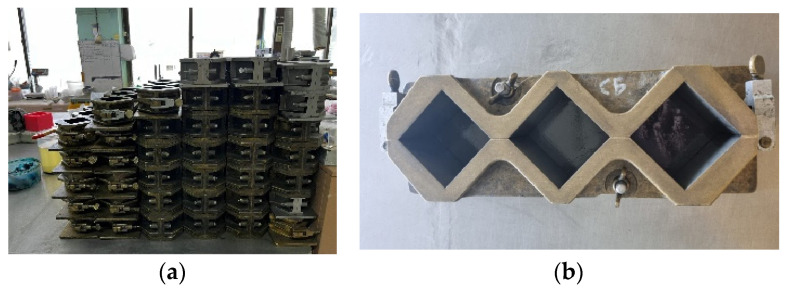
The mold used for preparing the mortar specimens and the specimens. Mortar moulding, (**a**) side view; (**b**) top view.

Specimen preparation was conducted in compliance with the relevant testing standards [18]. According to the standard, the production of six mortar cubes with dimensions of 5 × 5 × 5 cm^3^ requires 500 g of cement, 1375 g of quartz sand, and the prescribed amount of mixing water. This volume represents the amount required for a single mixing process. Upon completion of material batching, the cement paste and mortar mixtures were prepared using a laboratory mortar mixer (Controls Inc., Tucker, GA, USA) to ensure uniform blending and compliance with standard mixing procedures. Figure 2 illustrates the mold used for preparing the mortar specimens.

### 2.1. Physical Test

The fineness of both CTP and the cement was determined using the Blaine air permeability test with an automatic Blaine apparatus (Imtek Engineering, Schönwalde-Glien, Germany). Before testing, the sieved materials were ensured to be dry and free from agglomeration. The prepared sample was then carefully introduced into the permeability cell, with light tapping applied to eliminate any entrapped air voids. The mass of the filled cell was recorded using a digital balance (Sartorius, Göttingen, Germany).

The automatic Blaine apparatus was activated and allowed to complete its self-calibration process. The appropriate testing method was selected in accordance with the instrument’s operational protocol. The permeability cell was subsequently mounted into the chamber and secured until the indicator signaled proper locking. The automatic Blaine apparatus then measured the air-permeability flow time and automatically computed the specific surface area, expressed in cm^2^/g, as required by the test standard. The fineness values were recorded for all samples.

The specific gravity (SG) of the cement and CTP was determined using the Le Chatelier flask (Testmak, Ankara, Turkey) in accordance with standard procedures. The test is based on measuring the displacement of kerosene after a known mass of powder is introduced. Before testing, the flask was cleaned and dried, then filled with kerosene to a level between 0 and 1 mL, and the initial reading (V_1_) was recorded. Approximately 64 g of the powder sample, as typically required by the standard, was weighed and gradually introduced into the flask through a funnel to minimize splashing and prevent air entrapment. The flask was placed in a temperature-controlled water bath (20–27 °C) until both the temperature and liquid level stabilized. The final kerosene volume (V_2_) was then recorded, and the specific gravity was computed based on the observed displacement.

The normal consistency of cement was determined using a Vicat apparatus (Humboldt, Elgin, IL, USA) to identify the amount of water required for the cement paste to reach a specified level of workability. A total of 500 g of cement was mixed with a predetermined amount of water using a mechanical mixer until a homogeneous paste was obtained. The paste was then placed into a conical mold, and the surface was leveled carefully without applying additional pressure.

The filled mold was positioned under the Vicat apparatus, and the plunger was allowed to penetrate the paste. The test was completed within 30 s after mixing, under vibration-free conditions. The paste was considered to have normal consistency when the penetration depth reached 10 ± 1 mm from the original surface. If the requirement was not met, the water content was adjusted, and the test was repeated until the specified consistency was achieved.

The initial and final setting times of cement paste were determined using the Vicat apparatus to evaluate the onset and completion of hardening. The initial setting time indicates when the paste begins to lose its plasticity, while the final setting time represents the attainment of initial rigidity. These parameters are essential for assessing cement quality and ensuring adequate working time during mixing and application. Cement paste was prepared by mixing 650 g of binder with water corresponding to the normal consistency. For blended mixtures, the binder composition was adjusted according to the substitution percentage while maintaining a constant total mass of 650 g. The paste was mixed until homogeneous, molded into a conical ring, and its surface carefully leveled. The specimen was then stored in a curing chamber for 30 min. Penetration tests were carried out by allowing the Vicat needle to penetrate the paste for 30 s at different locations. The initial setting time was defined when the penetration reached 25 mm, while the final setting time was determined when the needle no longer left an impression on the surface of the specimen.

The mortar slump flow test was conducted to evaluate the workability of the mixture during casting and molding processes. The test was performed using a flow table in accordance with ASTM C305 [19] to determine the optimum water content required to achieve adequate workability, as it is strongly influenced by the water content of the cement paste. The procedure began by mixing distilled water with 500 g of cement, followed by resting for 30 s and mixing at 140 rpm for 30 s. Subsequently, 1375 g of quartz sand was added and mixed at 285 rpm for 30 s. The mixing was paused for 15 s and allowed to rest for 75 s, after which it was resumed for 60 s at 285 rpm until a homogeneous mortar was obtained. The mortar was then placed into a flow mold in two layers, each compacted appropriately, and the surface was leveled. The mold was lifted vertically, and the flow table was dropped 25 times. The spread diameter was measured in four directions, and the flow value was calculated accordingly. The acceptable flow range for normal consistency mortar is typically between 110 ± 5%, indicating adequate workability for practical applications.

### 2.2. Chemical Test

**Figure 3:**
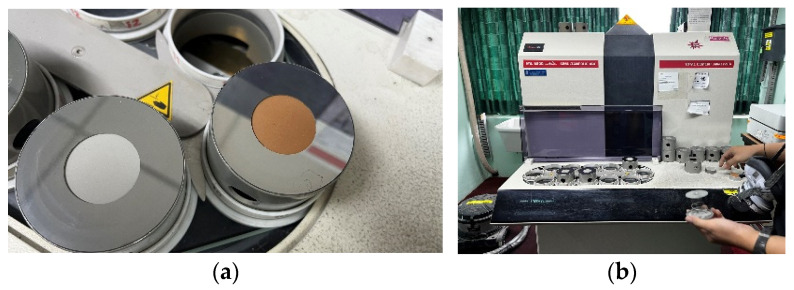
Chemical analysis of cement and CTP was conducted using X-ray fluorescence (XRF). (**a**) CTP in cylinder moulding; (**b**) analysed by XRF.

The chemical composition of the cement and CTP was analyzed using X-Ray Fluorescence (XRF) (Thermo Electron Engineering, Altrincham, UK). The XRF is located in a closed room at around 25 °C and 77% humidity. Before testing, both materials were oven-dried to remove residual moisture and subsequently ground until passing a fine sieve (<75 µm). When required, a small amount of binder was added to the powdered sample, after which it was compacted using a hydraulic press to produce a dense, smooth-surfaced pellet suitable for XRF measurements. Figure 3 illustrates that process. The pellet was then placed in the sample holder, and the XRF spectrometer irradiated it with X-rays. The emitted fluorescence signals were recorded and processed by the XRF. Based on the characteristic energies or wavelengths of these signals, the XRF automatically identified the elemental constituents, and the associated software quantified the major oxide concentrations in weight percent. The analytical results were displayed through the instrument’s computer interface.

### 2.3. Mechanical Test

Curing was performed by submerging the specimens in water maintained at controlled temperature and humidity conditions until the designated testing age. The water temperature is 23 ± 2 °C, and the humidity is not less than 50%. According to ASTM C109 [18], specimens must be removed from the curing bath immediately before conducting compression testing, surface-dried, and tested without delay to avoid moisture loss or other conditions that may influence the compressive strength results.

The compressive strength test was conducted to determine the load-bearing capacity of the mortar under axial compression. First, 241 mL of distilled water and 500 g of cement mixture are substituted with CTP. were added to the mixer bowl and allowed to rest for 30 s, followed by mixing at 140 rpm for 30 s. Thereafter, 1375 g of quartz sand was added, and mixing continued at 285 rpm for 30 s. The mixture was then paused for 15 s, rested for an additional 75 s, and subsequently mixed again for 60 s at 285 rpm to achieve uniformity [20].

Workability was assessed using the flow table test. The mortar was required to spread to 1–1.5 times the diameter of the mold to meet the slump-flow criteria. Mortar that satisfied this requirement was cast into 5 × 5 × 5 cm^3^ cube molds and subsequently cured through water immersion until the testing age.

(1) $$fc^{\prime }=\frac{P}{A}$$

**Figure 4:**
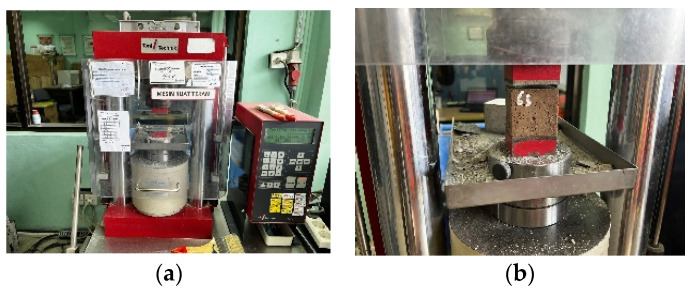
Illustrates the compressive strength test of mortar specimens conducted using a compression testing machine. Compression machine, (**a**) complete view; (**b**) process of mortar testing.

In Equation (1), fc′ is the compressive strength of mortar (MPa), P is the maximum applied load (N), and A is the loaded area (mm^2^). For a 50 mm mortar cube, A = 50 × 50 = 2500 mm^2^ [18]. Figure 4 illustrates the compressive strength test of mortar specimens conducted using a compression testing machine.

## 3. Results

### 3.1. Physical Test Result

The fineness characteristics of the cement and CTP were quantified using the Blaine air-permeability method. In comparison, CTP subjected to sequential grinding and sieving, consistently exceeded the minimum fineness requirement specified is 2800 cm^2^/g [17]. Extended milling further enhanced the specific surface area of the ceramic powder to 4871 cm^2^/g, exceeding that of the cement. This higher fineness indicates an increased surface area available for hydration reactions, suggesting enhanced reactivity of the CTP when incorporated as a cementitious material. Dicalcium Silicate in the cement will react with water to form Calcium Silicate Hydrate and Calcium Hydroxide. Further reaction then, Calcium hydroxide reacts with Silicate Oxide to form Calcium Silicate Hydroxide again. The reaction processes are shown below.

2C_2_S + 4H_2_O → C_3_S_2_H_3_ + Ca(OH)_2_

SiO_2_ + Ca(OH)_2_ → C-S-H

The specific gravity of the cement and CTP was calculated as 2.92–2.67 g·cm^−3^. While the cement satisfies the SNI 2531:2015 [21] requirement (2.90–3.15 g·cm^−3^), the CTP falls below the specified range. This discrepancy in density is critical for mortar mix design, as the lower specific gravity of the CTP may alter volumetric proportions, potentially reducing mixture homogeneity and contributing to the observed decline in compressive strength at higher replacement levels.

**Figure 5:**
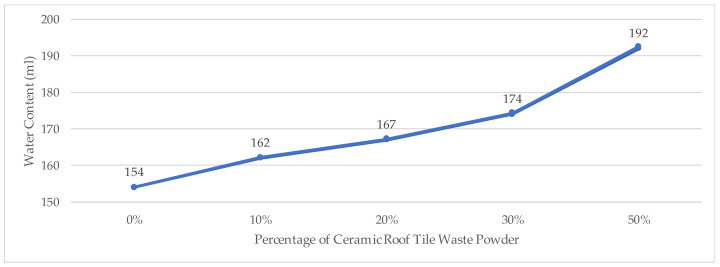
Normal Consistency Test.

Based on the experimental results, an increasing proportion of CTP substitution in the mortar mixture led to a higher water demand. As the replacement level increased, the normal consistency value consistently rose, indicating reduced workability at a constant water content. This behavior is attributed to the incorporation of CTP derived primarily from clay minerals, particularly kaolinite and montmorillonite [22]. Consequently, higher substitution levels of CTP require additional water to achieve normal consistency. The normal consistency results showing the water demand at different levels of CTP substitution are presented in Figure 5.

**Figure 6:**
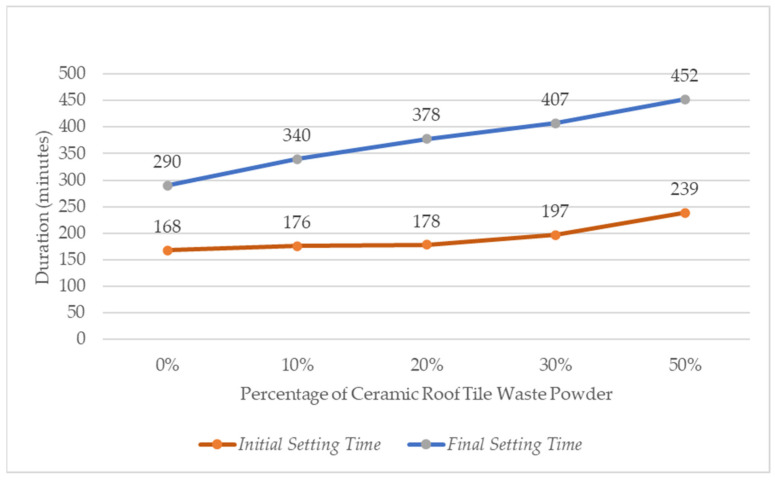
Comparison of Setting Time Test Based on the Percentage of CTP Substitution.

The test results indicate that the addition of CTP increases the setting time of cement paste. The control sample (0%) exhibited an initial setting time of 168 min and a final setting time of 290 min. With increasing substitution levels up to 50%, the initial setting time increased to 239 min, while the final setting time reached 452 min. This trend shows that higher percentages of waste powder lead to longer setting times. The delay in setting time is attributed to several factors, including the dilution effect caused by the reduction in key cement compounds such as C_3_S (Three Calcium Silicate) and C_2_S (Dicalcium Silicate), as well as the increased water demand observed in the normal consistency test. In addition, the pozzolanic nature of the waste material, which reacts more slowly than cement hydration, contributes to the slower development of the binding structure [23]. Despite the increase, the initial setting time still complies with the requirements of SNI 15-2049-2004 (45–375 min), indicating that the material remains suitable for practical applications. The trend of the test results is presented in Figure 6.

Figure 6 shows that both initial and final setting times increase with higher CTP substitution. The longest initial setting time occurred at 50% replacement (239 min), representing an increase of 5–42% compared to the control mixture. A similar trend was observed for final setting time, which increased by 17–56%, with the maximum value reaching 452 min at 50% substitution. As the CTP content increased, both initial and final setting times were prolonged, which is consistent with the reduced slump flow observed at higher replacement levels. The increased water demand and delayed hydration hinder early strength development, while the potential contribution to compressive strength is shifted to later ages through slower pozzolanic reaction.

The slump flow test was employed to evaluate mortar workability during casting and application, as this parameter directly affects mixture homogeneity and hardened density. The control mixture (0% CTP), referred to as BLANK, achieved this requirement at a water content of 241 mL, confirming satisfactory workability under standard conditions and serving as the reference for subsequent mixtures.

**Figure 7:**
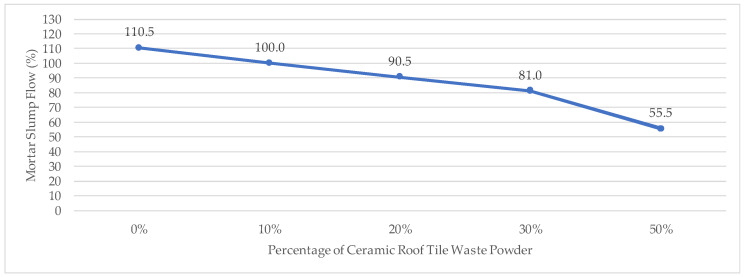
Slump Flow.

A systematic variation in slump flow was observed with increasing levels of CTP substitution. The progressive change in flow behavior indicates that workability is strongly influenced by the incorporation of CTP. This behavior can be attributed to the finer particle size and higher specific surface area of the ceramic powder, which increases water absorption and interparticle friction. Consequently, reduced flowability at higher replacement levels may contribute to delayed setting behavior and non-uniform compaction, which are consistent with the observed trends in setting time and compressive strength development. Detailed slump flow results and corresponding trends are illustrated in Figure 7.

Based on the slump flow test results, the control mortar (blank) with 100% PCC cement and 241 mL of water achieved a flow value of 110.5 mm, which falls within the required range (110 ± 5 mm) and can be used as a reference for workability. With the addition of CTP, the flow value decreased to 100 mm (10%), 90.5 mm (20%), 81 mm (30%), and 55.5 mm (50%). Overall, a consistent decreasing trend was observed, with reductions of 9.5%, 18.1%, 26.7%, and 49.8%, respectively, indicating that higher substitution levels result in lower mortar workability.

This reduction is attributed to the physical characteristics of the waste powder, including higher water absorption and specific surface area, which reduce the amount of free water available for lubrication between particles.

The influence of particle size distribution and surface texture increases internal friction within the mix [24]. Furthermore, previous studies [15] indicate that the water–cement ratio significantly affects pore structure. Therefore, increasing water content is not recommended, and the use of chemical admixtures such as plasticizers is preferred to maintain workability without compromising strength and durability.

### 3.2. Chemical Composition Result

**Figure 8:**
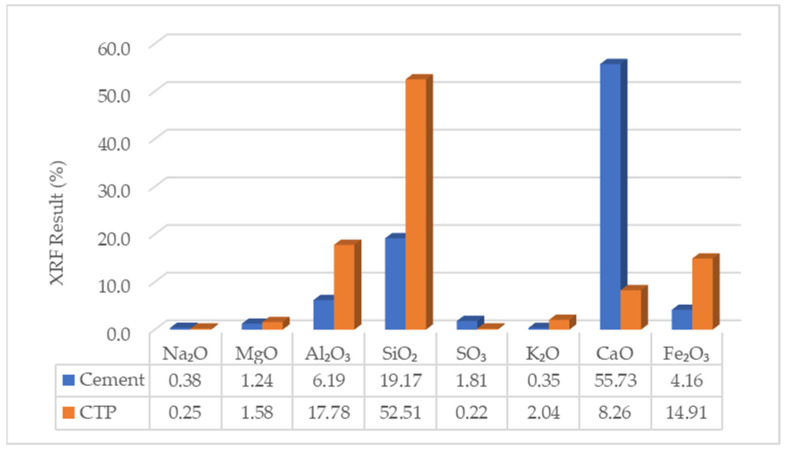
The chemical content of cement and CTP.

The results of the chemical composition analysis using X-ray fluorescence (XRF) are presented in Figure 8. Based on the chemical composition results of the cement, other main chemical compounds, namely C_3_S, C_2_S, C_3_A, and C_4_AF, can be determined using calculation formulas in accordance with SNI 15-2049-2004 on Portland cement. The results of the chemical content of cement are presented in Table 2. The other parts, which are not stated in Figure 8 and Table 2, include the insoluble part, Loss of Ignition, and other oxides, i.e., TiO_2_, V_2_O_5_.

Chemically, the CTP satisfies the requirements of a Class N pozzolan based on its high combined SiO_2_ + Al_2_O_3_ + Fe_2_O_3_ content. Its contribution to compressive strength is therefore indirect, occurring through delayed pozzolanic reactions that generate additional C–S–H gel, refine pore structure, and enhance strength at extended curing ages. As a result, the influence of CTP on compressive strength becomes more evident at later ages rather than during early hydration.

### 3.3. Mechanical Test Results

**Figure 9:**
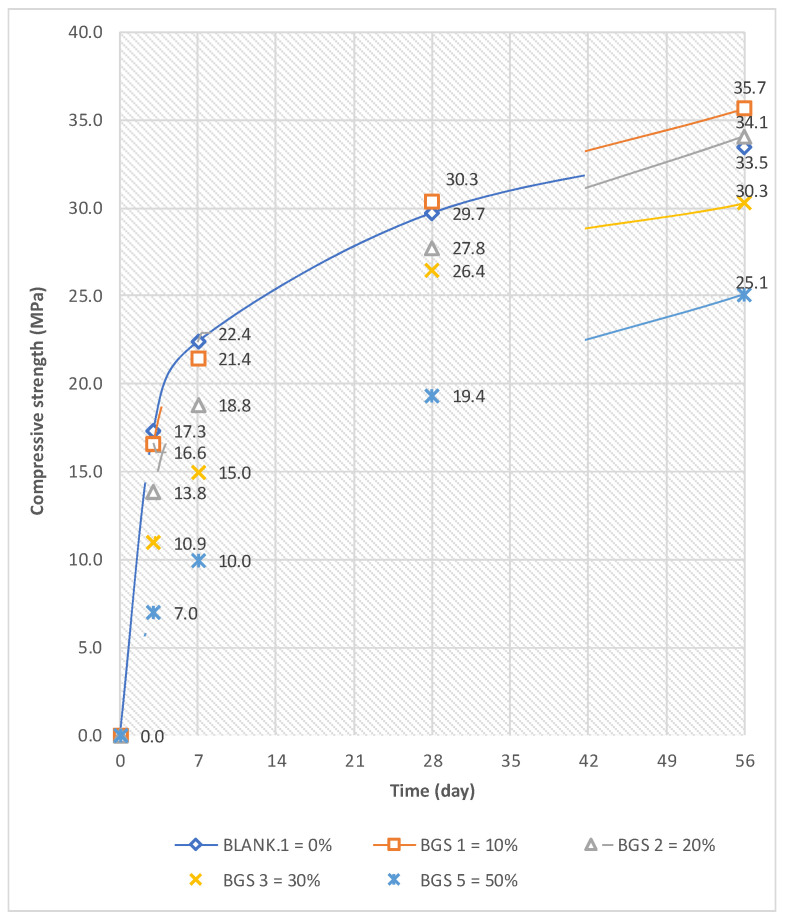
Average of Compressive Strength of Mortar.

The compressive strength results indicate that increasing CTP substitution generally reduces early-age strength (3 and 7 days), while strength development at later ages becomes more pronounced. This trend is consistent with the observed reduction in slump flow and the prolongation of initial and final setting times at higher replacement levels, which reflect increased water demand and delayed early hydration. At replacement levels of 10–20%, the 28-day compressive strength remains comparable to the control mixture, suggesting that the filler effect and improved particle packing partially compensate for cement dilution. However, higher substitution levels (≥30%) result in a notable reduction in early and intermediate strength due to limited clinker content and slower reaction kinetics. At extended curing ages (56 days), strength recovery is observed across all mixtures, indicating the contribution of delayed pozzolanic reactions. This explanation is illustrated in Figure 9. In Figure 9, Blank.1 means that the mortar mixture is without the addition of ceramic roof-tile waste powder. Moreover, BGS 1, BGS 2, BGS 3, and BGS 5 represent the mortar mixture with the content of ceramic roof-tile waste powder of 10, 20, 30, and 50% by weight of cement.

## 4. Discussion

The experimental results demonstrate that the incorporation of CTP significantly influences the physical, chemical, and mechanical behavior of mortar. From a physical perspective, the CTP exhibited a higher fineness (4871 cm^2^/g) compared to cement, indicating a greater specific surface area that enhances its reactivity potential. However, its lower specific gravity (2.67 g·cm^−3^) relative to cement (2.92 g·cm^−3^) affects the volumetric composition of the mixture, which may reduce homogeneity and contribute to strength reduction at higher substitution levels [4].

The higher fineness and porous nature of CTP also increase water demand, as reflected in the normal consistency and slump flow results. A continuous decrease in flowability with increasing substitution levels confirms that workability is strongly governed by particle characteristics, particularly specific surface area and water absorption capacity. The increased surface area requires more water for wetting, while the porous structure absorbs part of the mixing water, thereby reducing the amount of free water available for lubrication. This leads to higher internal friction within the mixture and reduced flowability. Consequently, higher substitution levels may adversely affect compaction quality, resulting in increased void content and lower density of the hardened mortar [9].

The influence of CTP is also evident in the setting behavior of cement paste. Both initial and final setting times increase with higher replacement levels, which can be attributed to the dilution of reactive clinker phases such as C_3_S and C_2_S, as well as the higher water demand and slower reactivity of the ceramic material. In addition, the reduced availability of calcium ions in the system delays the formation of hydration products. Although the setting time is prolonged, it still complies with standard requirements, indicating that the material remains suitable for construction applications. This retardation effect is closely associated with a slower early hydration rate, which consequently delays early-age strength development.

From a chemical perspective, the absence of C_3_S in the CTP explains its limited contribution to early-age strength, as this phase is primarily responsible for rapid strength development through early hydration. In contrast, the presence of C_2_S and the high contents of SiO_2_, Al_2_O_3_, and Fe_2_O_3_ indicate that the material exhibits pozzolanic characteristics. These oxides react with calcium hydroxide (Ca(OH)_2_) produced during cement hydration to form additional calcium silicate hydrate (C–S–H) gel. This secondary reaction contributes to long-term strength development and pore refinement. Therefore, the beneficial effects of CTP become more significant at later curing ages due to the gradual progression of pozzolanic reactions.

Compressive strength results, shown at early ages (3 and 7 days), all mixtures containing CTP exhibit lower strength than the control mix due to delayed hydration and reduced clinker content. However, at 28 days, mixtures with 10–20% replacement achieve comparable strength to the control, indicating that the filler effect and improved particle packing partially compensate for cement dilution. At higher replacement levels (≥30%), the reduction in strength becomes more pronounced due to insufficient cementitious compounds and slower reaction kinetics. Nevertheless, at 56 days, a noticeable strength recovery is observed, confirming the contribution of long-term pozzolanic reactions and microstructural densification.

Overall, the results indicate that CTP can be effectively utilized as a supplementary cementitious material, particularly at moderate replacement levels (10–20%). Its performance is governed by a combination of physical properties (fineness, particle morphology, and density), chemical composition (pozzolanic activity), and its influence on fresh properties such as workability and setting time. Importantly, there exists a trade-off between early-age strength reduction and long-term performance enhancement, which highlights the need for optimizing the replacement level. These findings are consistent with previous studies on ceramic-based waste materials, emphasizing that proper proportioning is essential to maximize sustainability benefits while maintaining mechanical performance.

## 5. Conclusions

This study demonstrates that the fineness of CTP plays a crucial role in determining its performance as a cement substitution material in mortar. Higher fineness improves particle packing; however, it also increases water demand, which directly affects mortar workability and prolongs setting time. As the substitution level of CTP increases, mortar workability decreases, and early-age compressive strength is reduced due to delayed hydration. Nevertheless, at moderate substitution levels (10–20%), the 28-day compressive strength remains comparable to that of the control mortar, which can be attributed to the filler effect and improved microstructural densification. After the mortar specimen with CTP was 56 days old, an increase in compressive strength was observed in all mixtures, indicating the contribution of delayed pozzolanic reactions. The mortars with CTP 10% and 20% have a compressive strength at 56 days, which is higher than that of the mortar without CTP. The compressive strength of mortar with 10% and 20% CTP increases by around 6.5% and 1.7% compared to mortar without CTP. This behavior is associated with the slow hydration of the C_2_S phase, which results in strength development becoming more pronounced after 28 days.

## Figures and Tables

**Table 1:** The Mixture of Mortar.

Material	Content of CTP (%)				
	0	10	20	30	50
Cement (gr)	500	450	400	350	250
Sand (gr)	1375	1375	1375	1375	1375
Water (gr)	241	241	241	241	241
CTP (gr)	0	50	100	150	250

**Table 2:** The Main Compound of Cement.

Main Compound	Value (%)
C_3_S	28.5
C_2_S	22.46
C_3_A	9.36
C_4_AF	12.66

## Data Availability

The original contributions presented in this study are included in the article. Further inquiries can be directed to the corresponding author.
